# Exploring the Evidence for an Immunomodulatory Role of Vitamin D in Juvenile and Adult Rheumatic Disease

**DOI:** 10.3389/fimmu.2020.616483

**Published:** 2021-02-18

**Authors:** Jiaqi Zou, Clare Thornton, Emma S. Chambers, Elizabeth C. Rosser, Coziana Ciurtin

**Affiliations:** ^1^ Centre for Rheumatology Research, Division of Medicine, University College London, London, United Kingdom; ^2^ Department of Rheumatology (Metabolic Bone Diseases), University College London Hospital NHS Foundation Trust, London, United Kingdom; ^3^ Centre for Immunobiology, Blizard Institute, Queen Mary University of London, London, United Kingdom; ^4^ Centre for Adolescent Rheumatology Versus Arthritis at University College London, University College London and Great Ormond Street Hospitals, London, United Kingdom

**Keywords:** vitamin D, rheumatoid arthritis, juvenile idiopathic arthritis, systemic lupus erythematosus, immunomodulatory, autoimmunity

## Abstract

Vitamin D is synthesized in the skin following exposure to UVB radiation or is directly absorbed from the diet. Following hydroxylation in the liver and kidneys, vitamin D becomes its bioactive form, 1,25(OH)_2_D, which has been described to have potent immunomodulatory capacity. This review will focus on the effect of vitamin D in modulating the dysregulated immune system of autoimmune rheumatic diseases (ARD) patients across age, in particular in arthritis (rheumatoid arthritis and juvenile idiopathic arthritis), and systemic lupus erythematosus (with adult and juvenile onset). As well as delineating the impact of vitamin D on the innate and adaptive immune functions associated with each disease pathology, this review will also summarize and evaluate studies that link vitamin D status with disease prevalence, and supplementation studies that examine the potential benefits of vitamin D on disease outcomes. Exploring this evidence reveals that better designed randomized controlled studies are required to clarify the impact of vitamin D supplementation on ARD outcomes and general health. Considering the accessibility and affordability of vitamin D as a therapeutic option, there is a major unmet need for evidence-based treatment recommendations for the use of vitamin D in this patient population.

## Introduction

**Figure 1:**
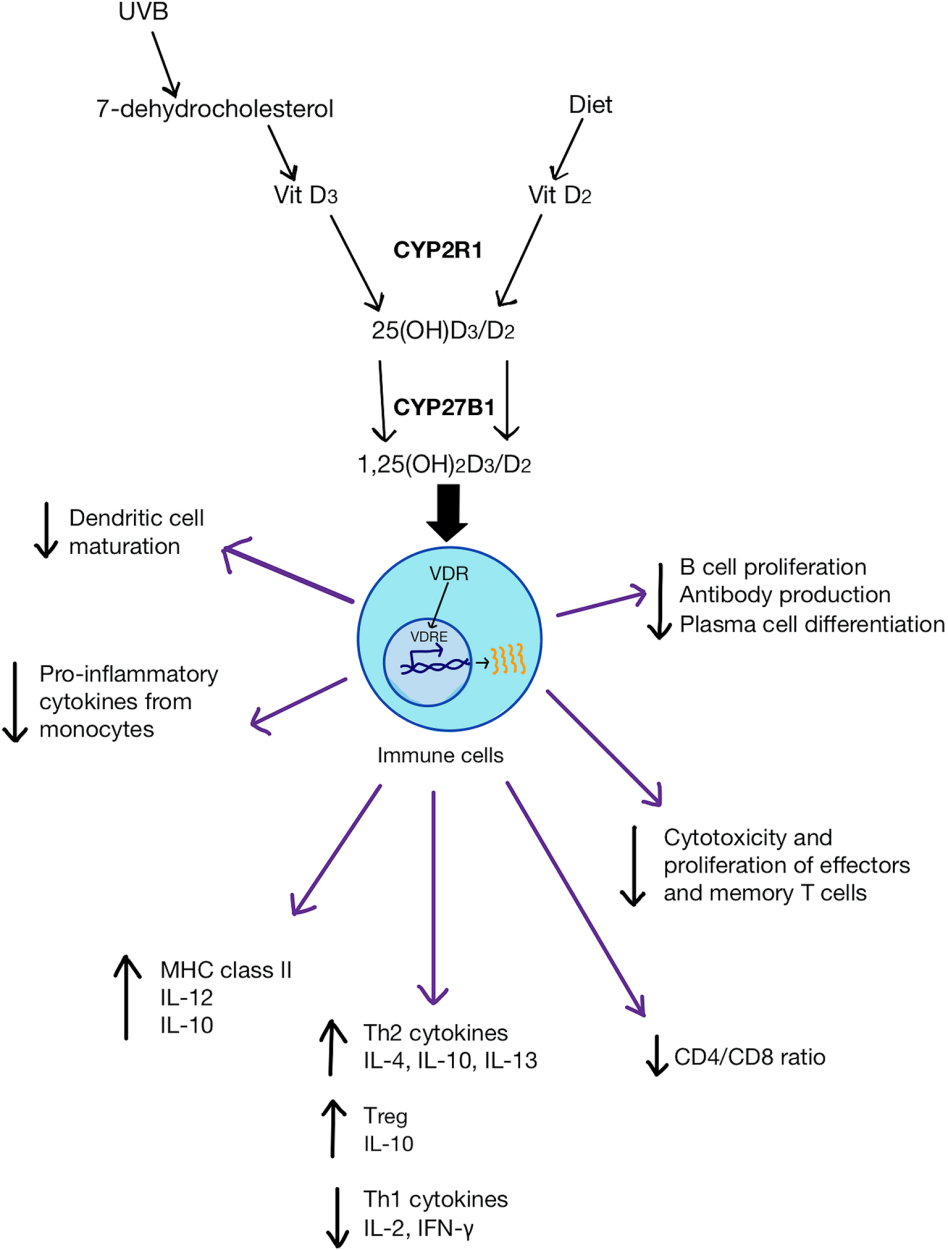
Vitamin D (both D_3 _and D_2_) metabolic pathway and its physiological impact on immune cells. D_3_ is synthesized from either 7-dehydrocholesterol from the skin *via* UVB activation or through the diet. D_2_ is obtained from the diet. CYP2R1 enzyme converts D_3_/D_2_ to 25(OH)D_3_/D_2_ in the liver. CYP27B1 transforms 25(OH)D_3_/D_2_ into 1,25(OH)_2_D_3_/D_2_ in the kidneys. 1,25(OH)_2_D binds to VDR in immune cells which then bind to VDRE on genes to change gene expression. 1,25(OH)_2_D can inhibit dendritic cell maturation, promote monocyte proliferation and differentiation into macrophages. It can also increase anti-inflammatory cytokines and the frequency of T_regs_, as well as reduce pro-inflammatory Th1 cytokines. 1,25(OH)_2_D can also reduce the ratio of CD4/CD8 and inhibit pro-inflammatory T cell differentiation and suppress antibody production by B cells.

Vitamin D is an essential micronutrient synthesized in the body or obtained from the diet. In recent decades, vitamin D deficiency (< 50 nmol/L) and insufficiency (50–75 nmol/L) has emerged as a widespread health problem (**Table 1**) (1, 2). According to available evidence, less than 50% of the global population have adequate vitamin D levels in their blood (3). The predominant bioactive form of vitamin D is cholecalciferol (vitamin D_3_), which is synthesized in the skin from 7-dehydrocholesterol following exposure to sunlight (UVB radiation). Other forms of vitamin D (mainly as ergocalciferol or vitamin D_2_) can also be absorbed from various foods e.g., oily fish, red meat, liver, egg yolks, fortified cereals, and specific supplements e.g., fish oil. Both cholecalciferol and ergocalciferol are converted *via* a two-step activation process (**Figure 1**). Firstly, cholecalciferol and ergocalciferol are hydroxylated in the liver and converted into the inactive metabolites 25-hydroxyvitamin D [25(OH)D_3_ (calcifediol) and 25(OH)D_2_ (ercalcidiol)], respectively. Secondly, calcifediol and ercalcidiol are hydroxylated to the bioactive form 1,25(OH)_2_D_3_ (calcitriol) and 1,25(OH)_2_D_2_ (ercalcitriol), respectively, in the kidneys. Much like other steroid hormones, both calcitriol and ercalcitriol bind to the vitamin D receptors (VDR) within cells, albeit ercalcitriol with a much lower affinity. VDR then heterodimerise with retinoid X receptors (RXR) and the complex translocates into the nucleus (4). This heterodimer in turn binds to vitamin D receptor elements (VDRE) present in various cell genes, altering gene expression (4). Two types of vitamin D supplementation are available, vitamin D_2_ and D_3_.

**Table 1:** Definition of vitamin D status. 1 nmol/L = 0.4 ng/mL.

Vitamin D status	Concentration of serum 25(OH)D
**Vitamin D deficiency**	<50 nmol/L
**Vitamin D insufficiency**	50–75 nmol/L
**Vitamin D sufficiency**	>75 nmol/L

The classical role of bioactive vitamin D is to promote intestinal and renal calcium absorption, maintaining a very precise regulation of calcium transfer between bones and blood stream. Positive calcium balance is characterized by net bone formation, either as bone growth or repair; while in negative calcium balance, vitamin D promotes bone resorption and inhibits bone mineralization to maintain serum calcium levels (5). As a consequence, vitamin D has an active role in the prevention of bone fractures associated with low bone density/osteoporosis, eliciting fracture reduction effect from a minimum serum concentration of 74 nmol/L, which is now widely accepted as lower limit of optimal vitamin D level (**Table 1**) (6). Following the observation that VDRs are also expressed by immune cells, endothelial cells, and vascular smooth muscle cells (7–9), there is now a wealth of studies demonstrating that vitamin D has an additional immunomodulatory role, as well as implications in cardio-vascular health. Recent research has linked vitamin D deficiency to the pathogenesis of various immune-mediated inflammatory diseases in both children and adults (10, 11), Importantly, there is some evidence that vitamin D deficiency may act as an environmental trigger for autoimmune disease development (12, 13), and in particular autoimmune rheumatic diseases (ARD) (14, 15), with some studies suggesting that there may be some therapeutic benefit of vitamin D supplementation in these diseases (13, 16).

In this review, we will discuss the evidence for an immunomodulatory effect of vitamin D on the two of the most prototypical ARD across age; rheumatoid arthritis (RA) and juvenile idiopathic arthritis (JIA) and juvenile and adult-onset systemic lupus erythematosus (SLE). Although, exploring the immunomodulatory role of vitamin D in the process of atherosclerosis which is a common co-morbidity associated with chronic inflammation as seen in various ARDs is beyond the scope of this review, it is worth mentioning the protective effects of vitamin D on endothelial activation and dysfunction, through inhibition of cyclooxygenase 2 and cellular/platelets adhesion molecules expression, as well as pro-inflammatory cytokines synthesis, therefore minimizing the inflammatory processes contributing to atherosclerosis plaque formation (17, 18).

## Immune Dysregulation in Rheumatoid Arthritis and Juvenile Idiopathic Arthritis

Although RA and JIA are completely different diseases, they are both characterized by immune-mediated joint inflammation. In both diseases, autoimmune arthritis is characterized by inflammation of the synovial membrane, which involves the proliferation of synoviocytes and invasion of inflammatory immune cells into the synovium. This leads to synovial membrane thickening, which, if left untreated, ultimately leads to irreversible articular cartilage destruction and bone erosions (19). In adults, RA is one of the most common chronic rheumatic diseases, affecting 0.5–1% of the global population (20). In children, autoimmune inflammatory arthritis is classified under the umbrella term of JIA and is also one of the most common rheumatic diseases in children and adolescents. JIA terminology encompasses many types of arthritis, which are pathologically distinct from RA with the exception of the rheumatoid factor (RF) positive polyarticular JIA phenotype, present in less than 10% JIA patients which is clinically, serologically and genetically similar to the RA phenotype in adults (21, 22). For simplicity, in this review, we will focus on studies in patients with oligo-articular JIA (oligo-JIA), which is the most common phenotype in children, and polyarticular-JIA (poly-JIA), where, as described above, there is some overlap with the pathogenesis of RA (23). Disease activity in RA is measured by using the Disease Activity Score-28 (DAS28) index, which includes swollen and tender joint counts out of 28 designated joints, ESR or CRP levels, and subjective rating of global health measured on a 1–10 visual analogue scale (VAS). For JIA, the most used disease activity measure is the Juvenile Arthritis Disease Activity Score (JADAS), which assesses a variable number of joints (10, 27, or 77), as well as physician and patient/parent VAS rating of disease activity and inflammation (ESR).

Although fundamentally different diseases, oligo-JIA, poly-JIA, and RA share many immunopathogenic features that lead to synovitis, including being characterized by a strong pro-inflammatory cytokine signature. Activated myeloid cells produce increased levels of TNF-α, IL-6, IL-1β at the inflamed site, and this is reflected in the shared therapeutic approaches of blocking these cytokines in both RA and JIA (24–26). There is also an imbalance of T-helper 1 (Th1) and T-helper 17 (Th17) cells compared to T helper 2 (Th2) and regulatory T (T_reg_ cells) (27), and both RA and JIA have been postulated to be mediated by both the type 1 cytokines such as IFN-γ produced and IL-17 (28). Collectively, TNF-α, IL-6, IL-1β, and IL-17 form positive feedback loops increasing the production of these potent inflammatory cytokines, promoting synovial inflammation, and bone resorption, which characterize both diseases. Additionally, in both diseases, B cells secrete autoantibodies, such as rheumatoid factors (RF), anti-cyclic citrullinated peptide (CCP) antibodies, and, in some oligo-JIA and poly-JIA patients, anti-nuclear antibodies (ANAs) (29, 30). B cells can also contribute to inflammation by producing chemokines and cytokines that trigger inflammation and synovial hyperplasia, and by presenting autoantigens to autoreactive T cells (31). It is thought that a complex interplay between genetic and environmental factors leads to this breakdown in immunological tolerance and thus synovial inflammation. Variants within HLA genes have been demonstrated to increase the risk of developing RA and JIA (32, 33), whilst environmental factors such as exposure to tobacco smoke and changes to the gut microbiota have been implicated in disease development (34, 35). In recent years, new evidence has emerged that vitamin D deficiency and insufficiency may also be an important environmental trigger in the onset of disease.

## Prevalence of Hypovitaminosis D and Its Impact on the Risk of Developing Rheumatoid Arthritis and Juvenile Idiopathic Arthritis

Numerous studies have investigated the relationship between vitamin D insufficiency/deficiency and autoimmune arthritis, establishing that RA patients present with lower vitamin D levels than healthy controls (36–39). A systematic review with 3,489 people from 24 reports found that RA patients had lower 25(OH)D levels than healthy controls with a mean difference of 16.52 nmol/L(= 6.6 ng/ml) (38). Likewise, the COMORA study measured vitamin D levels of 1,413 RA patients from 15 countries and reported that 63% of patients were either vitamin D insufficient or deficient (36). Similarly, in JIA patients, the evidence for hypovitaminosis D, or low vitamin D levels, is consistent across several studies (40–43). A scoping review in 2018 reported that 32/38 studies showed a high prevalence (84.2%) of suboptimal 25(OH)D levels (< 30ng/ml) in JIA patients (41). Similarly, a meta-analysis revealed widespread vitamin D deficiency status in JIA patients (up to 82%) (42). These data strongly suggest that children and adults with inflammatory arthritis have low concentrations of circulating vitamin D.

Another important consideration is whether individuals with low vitamin D levels are at risk for developing arthritis. There is controversial evidence regarding the impact of vitamin D intake on the risk of developing RA. The Iowa Women’s Health Study recorded the vitamin D intake of 29,368 women without RA at baseline with a self-reported questionnaire and followed them through 11 years (44). They found a negative correlation between vitamin D intake and RA risk (relative risk RR = 0.72). Conversely, the Nurses’ Health Study reported no relationship between vitamin D intake and risk of RA (45). Yet, interestingly, a meta-analysis of the Iowa and NHS cohorts found an overall significant relationship between RA risk and total vitamin D intake (diet and supplements) (RR = 0.758, p = 0.047) (46). Patients with the lowest vitamin D intake had a 24.2% higher risk of RA than those with the highest vitamin D intake. Together, these studies indicate that it is challenging to get an accurate picture of vitamin D intake and RA risk. This brings into question the validity of dietary intake assessments with food frequency questionnaires without measuring serum vitamin D levels. Limitations of these questionnaires include reporting bias and their closed and limited format. There are no similar studies for JIA.

## Vitamin D Levels and Disease Activity: Therapeutic Implications for Rheumatoid Arthritis and Juvenile Idiopathic Arthritis

Considering the evidence that both JIA and RA patients have low vitamin D levels, there is growing interest in understanding whether altering vitamin D levels can impact disease activity. For RA, most studies have concluded that serum 25(OH)D levels inversely correlated with the DAS28 index (36–38, 46) (**Table 2**). From the limited studies available in JIA, it remains controversial whether 25(OH)D levels correlate with disease activity. However, a number of studies found negative correlations between JIA disease activity and serum 25(OH)D levels, although it remains difficult to infer a causal relationship (43, 50, 51), as the majority of studies were cross-sectional. No causal relationship between vitamin D levels and disease activity can be inferred as the evidence from randomized controlled trials (RCTs) is lacking. The evidence for a link between vitamin D supplementation and RA disease activity is inconsistent. Some studies suggested a decrease in DAS28 following supplementation, whereas other studies reported no significant change in DAS28 or flare rate after supplementation (**Table 3**). Due to the variation in the type of vitamin D supplementation, dose, and duration, it is extremely difficult to compare these studies. The majority of studies supplemented vitamin D deficient patients only (52–55); two studies supplemented patients irrespective of serum levels (57), though in one study, 90% of the patients were vitamin D insufficient (58). For JIA, only one cross-sectional study has investigated the role of vitamin D supplementation in modulating disease activity and did not report any therapeutic benefits (58). Standardizing the method and amount of supplementation would allow more comparison across different studies and cohorts. Further studies are needed to clarify whether vitamin D has any therapeutic benefits for in RA or JIA.

**Table 2:** Impact of vitamin D concentration on RA and JIA outcomes.

Author et al., year	Country Type of study	Number of participants,F:M Age	Disease duration	Impact on disease outcomes
**RA**				
Hajjaj-Hassouni et al., 2017 (COMORA study) (36)	Asia, Europe, North America, South America, Africa Post-hoc analysis of a Cross-sectional study	1,413 RA patients 4.86:1 57.9 ± 12.8	8.3 (3.6–15.2) years	Serum 25(OH)D levels and DAS28 were inversely correlated (r = -0.104; p < 0.001)
Lin et al., 2016 (38)	Asia, Europe, North America, South America, Africa, 24 studies Systematic review and meta-analysis	3,489	N/A	Serum 25(OH)D levels and DAS28 were inversely correlated (r = -0.13; p = 0.02).
Song et al., 2012 (46)	Europe, North America, Middle East Systematic review and meta-analysis 8 studies	2,885 RA patients 1,084 controls	N/A	7 out of 8 studies reported an inverse relationship between vitamin D levels and DAS28
Lee and Bae, 2016 (37)	Asia, Europe, South America, Africa 15 Case-control studies	924 RA patients	N/A	Inverse correlation between vitamin D levels and DAS28 (r = -0.278, p < 0.001)
Higgins et al., 2013 (47)	UK Cross-sectional study	176 RA patients 3:1 64 (22–89)	12 (1–37) years	No significant correlation between vitamin D levels and DAS28 with or without GVAS. Significant inverse correlation between vitamin D levels and VAS-pain (r = -0.249, p = 0.013)
Pakchotanon et al., 2016 (48)	Thailand Cross-sectional	239 RA patients 6.9:1 58.85 ± 12.56	112.79 ± 203.03 months	No correlation between vitamin D levels and DAS28.
**JIA**				
Pelajo et al., 2012 (49)	US Cross-sectional study	154 JIA patients 1.56:1 10.6 ± 4.5	28 (6–66) months	No correlation between 25(OH)D levels and JADAS27 JADAS27 was higher in polyarticular JIA than oligoarticular JIA (p = 0.003)
Stagi et al., 2014 (43)	Italy Cross-sectional study	152 JIA patients 3.1:1 16.2 ± 7.2	129.5 ± 11.11 months	Patients with active disease and frequent relapses have significantly lower 25(OH)D levels than those without (p < 0.005)
Çomak et al., 2014 (50)	Turkey Cross-sectional	47 JIA patients 1.56:1 9.3 ± 3.9	28 months	Negative correlation between 25(OH)D levels and disease activity (r = -0.37, p = 0.01) JADAS27 is greater in patients with 25(OH)D levels <15 ng/ml than those >15 ng/ml (p = 0.003)
Shevchenko and Khadzhynova, 2019 (51)	Georgia Cross-sectional	69 JIA patients 1.9:1 10.7 ± 4.5	4.1 ± 1.1 years	A significant relationship between vitamin D level and number of active joints.

DAS28, disease activity score assessing 28 joints; JADAS 27, juvenile arthritis disease activity score assessing 27 joints; JIA, juvenile idiopathic arthritis; RA, rheumatoid arthritis; VAS, visual analogue scale; N/A, not available; 25(OH)D, 25-hydroxy vitamin D.

**Table 3:** Impact of vitamin D supplementation on RA and JIA outcomes.

Author et al., year	Country Type of study Study duration	Number of patients vs. control group (if any) F:M, Age (mean +/- SD)	Vitamin D dose	Impact on disease activity
**RA**				
Chandrashekara and Patted 2017 (52)	India; Randomized, open-label interventional study; 3 months	149 RA patients 15.6:1 49 ± 12.1	60,000 IU/week for 6 weeks then 60,000 IU/month for 1 month	Mean DAS28-CRP decreased significantly: 3.68 ± 0.93 to 3.08 ± 1.11 (p = 0.002)
Dehghan et al., 2014 (53)	Iran RCT 6 months	40 patients in the intervention arm vs. 40 in the placebo arm; 7:1 45 ± 8.66 vs. 42.7 ± 9.77 for case and control groups,	50,000 IU/week	No significant difference in DAS28 between intervention and placebo group. No significant difference in flare rate between intervention (17.5%) and placebo (27.5%) group.
Yang et al., 2015 (54)	China RCT 24 months	64 case group vs 88 control 6.8:1 44.2 ± 7.5 vs. 41.7 ± 8.6	Alfacalcidol 0.5 µg/day	No significant difference in disease recurrence (based on DAS28 values) between treatment and non-treatment group.
Hansen et al., 2014 (55)	US RCT 12 months	11 patients in the intervention arm vs. 11 in the placebo arm 0.83:1 63 ± 12 vs. 53 ± 11	Ergocalciferol 50,000 IU*3/week for 4 weeks, then 50,000 IU*2/month for 11 months	No significant difference in DAS28 between placebo and treatment group.
Franco et al., 2017 (56)	Iran, China, US Systematic review and meta-analysis	240 patients in the intervention arm vs. 410 patients in placebo arm	Various	No significant difference in DAS28 between placebo and treatment groups.
Adami et al., 2019 (57)	Italy Exploratory study 3 months	61 RA patients 4.4:1 58 ± 12	100,000 IU/month vit D_3_	Significantly decreased DAS28 scores in patients with 25(OH)D ≥20 ng/ml (p < 0.01) after 3 months. No significant decrease in patients with 25(OH)D <20 ng/ml after 3 months
**JIA**				
Tang et al., 2019 (58)	China Open-label, prospective RCT 24 weeks	18 patients in the intervention arm vs. 18 in the control arm 1.8:1 7.6 ± 4.3 vs 6.3 ± 2.9	2,000 IU/day vit D_3_	No significant decrease in disease activity

DAS28, disease activity score assessing 28 joints; DAS28-CRP, disease activity score assessing 28 joints including C-reactive protein as inflammatory marker; JIA, juvenile idiopathic arthritis; RA, rheumatoid arthritis; RCT, randomized controlled trial; 25(OH)D, 25-hydroxy vitamin D.

## Immunomodulatory Mechanisms for Vitamin D in Rheumatoid Arthritis and Juvenile Idiopathic Arthritis

**Figure 2:**
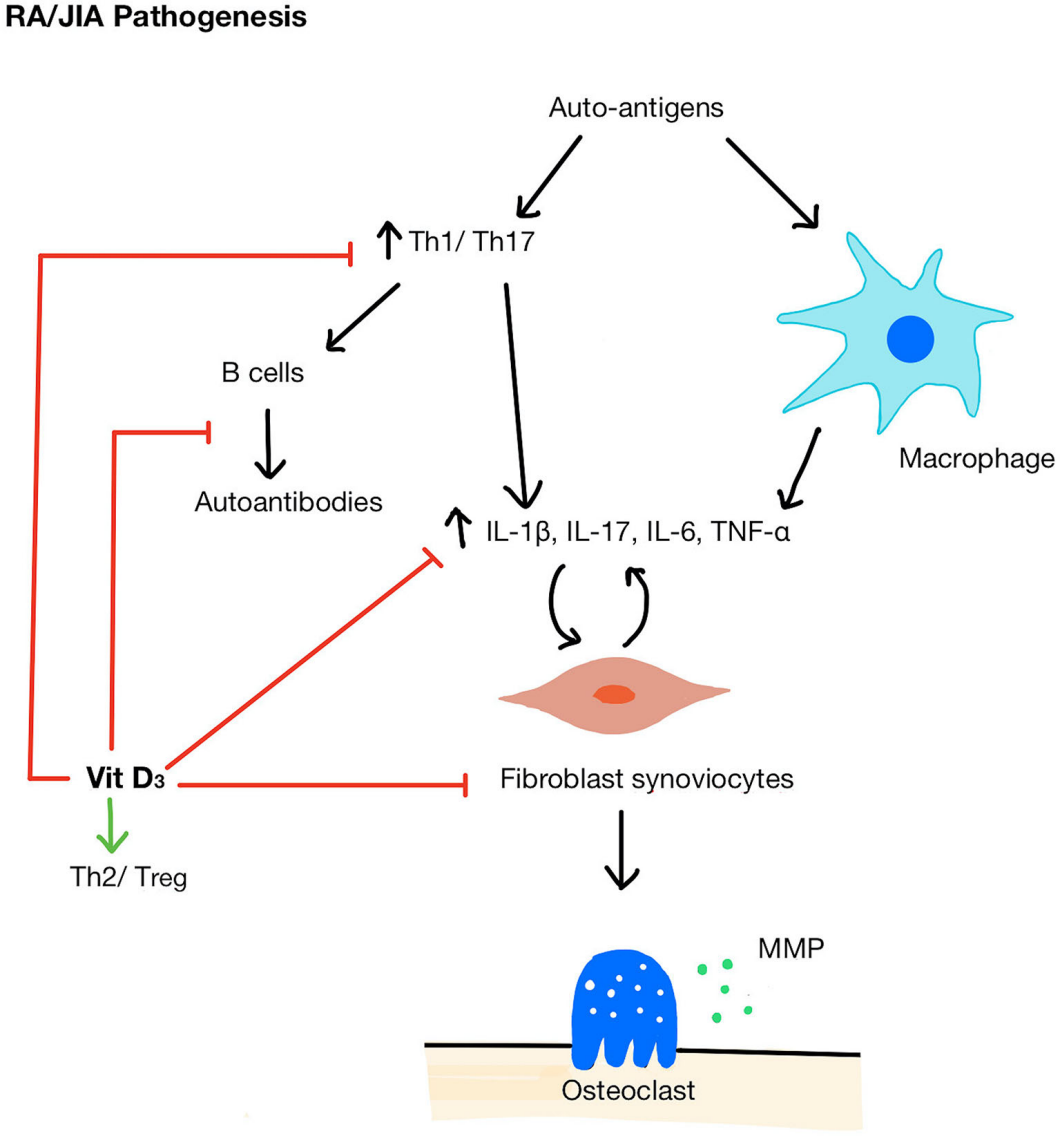
Pathogenesis of RA/JIA and mechanisms of vitamin D acting on immune cells. A breakdown in tolerance in RA/JIA leads to increased Th1/Th17 cells and promotes macrophages to produce pro-inflammatory cytokines (IL-1β, IL-17, IL-6, TNF-α) which in turn stimulates fibroblast synoviocyte proliferation. Synoviocytes then activate osteoclasts and secrete MMPs which break down bone and cartilage. Vitamin D shifts the balance of Th cells from Th1/Th17 to Th2/T_reg_, inhibit B cells and autoantibody production, reduces pro-inflammatory cytokines, and inhibits synoviocyte proliferation. MMP, matrix metallopeptidase.

There are several mechanisms by which vitamin D could modify inflammatory pathways in autoimmune inflammatory arthritis (**Figure 2**). For example, 1,25(OH)_2_D_3_ can influence myeloid-derived cytokine pathways. An *in vitro* study demonstrated that the exposure of RA fibroblast-like synviocytes to 1,25(OH)_2_D_3_ (in doses ranging from 0.1 to 100 nM, therefore equivalent to sub-physiological and optimal vitamin D serum concentrations) decreased TNF-α and IL-6 expression and at the same time, reduced osteoclastogenic RANKL (receptor activator of nuclear factor kappa-B ligand) levels relative to OPG (osteoprotegerin), an inhibitor of RANKL activity (59). *In vivo*, 1,25(OH)_2_D_3_ treatment at supra-physiological concentration (1 μmol/L) inhibited synoviocyte invasion and reduced IL-1β and matrix metalloprotease-1 (MMP-1) when measured *ex vivo* (60). Although the mechanism by which this suppression of cytokine production by synoviocytes remains unknown, 1,25(OH)_2_D_3_ was detected in the arthritic synovium and VDRs were found in rheumatoid synovial tissue suggesting that there may be direct suppression of cytokine gene expression in these cells (61). Interestingly, it has been suggested that 1,25(OH)_2_D_3_ can only act as negative feedback to reduce inflammatory responses when the immune system is activated (62), in which 1,25(OH)_2_D_3_ can promote synoviocyte apoptosis but only in the presence of TNF-α (63).

1,25(OH)_2_D_3_ can also alter T cell differentiation by promoting a shift of Th cells from Th1/Th17 profile (64, 65), which is pathogenic in both RA and JIA, to a Th2/T_reg_, profile, associated with anti-inflammatory properties (66). 1,25(OH)_2_D_3_ stimulates the expression of both IL-10^+^ and Foxp3^+^ T_reg_ cells in human T cell culture (67, 68). 1,25(OH)_2_D_3_ inhibited cytokines essential for Th1 and Th17 maturation (66) and upregulated the expression of transcription factors that induce Th2 cell development (69). Interestingly, in RA patients, serum 25(OH)D level was inversely correlated with IL-17 levels (70) and *in vitro* physiological doses of 1,25(OH)_2_D_3_ have been shown to decrease the IL-17 production by Th17 cells (71). Considering these complementary actions of immunosuppression, it has been suggested that that vitamin D may be more efficacious at suppressing immunological pathogenic mechanism when acting as an adjunct therapy. For example, Kim et al. found that 25(OH)D sufficient RA patients had greater DAS28 reduction in response to tocilizumab treatment (an IL-6 receptor antagonist) than 25(OH)D deficient patients and the difference persisted for 48 weeks (72). This study also showed that the addition of tocilizumab and 1,25(OH)_2_D_3_ *in vitro* suppressed the IL-17 production synergistically (72). A significant limitation of this study was that only baseline 25(OH)D level was recorded; it did not account for any changes in 25(OH)D levels in patients throughout the follow-up period. Furthermore, *in vitro*, 1,25(OH)_2_D_3_ at an optimal physiological concentration in combination with corticosteroids additively inhibited the TNF-α, IL-17, IL-6, and matrix metallopeptidase (MMP) production by synoviocytes co-cultured with T-cells (73). Notably, TNF-α could only be inhibited by the combination of both 1,25(OH)_2_D_3_ and corticosteroid and not by either alone, suggesting that vitamin D was beneficial only in combination with corticosteroid exposure. Although these *in vitro* experimental setting cannot replicate the pathological synovial environment of RA or JIA, they do provide evidence that vitamin D supplementation could be more efficacious at suppressing inflammatory mediators when given in combination with other therapeutic agents.

It is important to note that one study argued against the use of 1,25(OH)_2_D_3_ to treat active RA, by demonstrating that there was a difference in the effect of optimal physiological 1,25(OH)_2_D_3_ concentration on Th17 in serum compared to those in the synovial fluid (74). The authors proposed that 1,25(OH)_2_D_3_ had a lower inhibitory effect on Th17 in the synovial fluid because 1,25(OH)_2_D_3_ is less effective on the more committed T cell phenotypes present in the synovium compared to the naïve T cells in the serum. Thus, they questioned the efficacy of vitamin D as a potential treatment for active RA patients; rather, they proposed it can be used as a prophylactic measure to reduce the recurrence of flares in patients in remission. Future studies should investigate the reason behind the lack of synovial response to 1,25(OH)_2_D_3_ and explore ways to improve its sensitivity. At the same time, supplementation studies should be conducted in subjects at risk of RA or in the early stages of RA to explore vitamin D role in preventing abnormal immune activation (62).

## Immune Dysregulation in Systemic Lupus Erythematosus With Juvenile and Adult Onset

SLE is a severe autoimmune rheumatic disease that is characterized by chronic, multisystem inflammation (75). It has an unpredictable course, with disease flares alternating with periods of clinical remission (76). The disease is more prevalent in females than males and peaks at the time of puberty in girls. SLE occurs most commonly in patients of 15–44 years of age, although 15–20% of cases start in childhood or adolescence (in which case the disease is called juvenile SLE- JSLE) (77, 78). JSLE can often be more severe, involving life-threatening damage to the kidneys and central nervous system. SLE patients overall require long term and aggressive treatments, and the mortality is increased in JSLE compared with adult-onset SLE. The primary diagnosis for SLE and JSLE is made using a combination of characteristic clinical features and detection of autoimmune antibodies, such as ANA, and in particular, antibodies against double-stranded DNA (dsDNA) which are specific for SLE (79). In clinical settings and experimental studies, SLE disease activity is measured using the SLE disease activity index (SLEDAI) which is a global index comprising 24 clinical and laboratory variables (80).

SLE is primarily thought of as a type 1 IFN driven disease. Both JSLE and SLE patients have an increased IFN signature, defined as overexpression of IFN-inducible genes in monocytes and lymphocytes, when compared to healthy controls (81, 82). It is thought that endogenous nucleic acids released by apoptotic cells or historic viral infections lead to the production of IFN-α by plasmacytoid dendritic cells. IFN-α goes on to activate autoreactive B cells, T helper (Th) cells and cytotoxic T cells. These endogenous nucleic acids and apoptotic cells are then bound by anti-nuclear autoantibodies (ANAs), leading to immune complex deposition and epitope spreading. Self-antigens are also presented by mature dendritic cells (non-tolerogenic) (83, 84), which activate pro-inflammatory Th cells (83). Similarly to RA, HLA genetic variants and complement genes have been associated with SLE (85). Environmental factors such as UV light, Epstein-Barr virus infection and obesity could contribute to SLE predisposition. A key feature of lupus is the skin photosensitivity, which leads to many SLE patients avoiding exposure to sunlight, it is perhaps unsurprising that low vitamin D levels have been described in many lupus patients.

## Prevalence of Hypovitaminosis D and Its Impact on the Risk of Developing JSLE and Adult-Onset Systemic Lupus Erythematosus

The evidence for inadequate vitamin D levels in SLE is well documented. Recently, a systematic review further confirmed the high prevalence of vitamin D insufficiency in SLE (86). It evaluated 34 case-control studies consisting of 2265 SLE patients and concluded that serum 25(OH)D levels were significantly lower in SLE patients than controls (p < 0.00001). Other cross-sectional studies across Europe, South America, and Asia have reached similar conclusions that the overwhelming majority of SLE patients had insufficient vitamin D levels, which were significantly lower than healthy controls (87–89). As described above, the prevalence of hypovitaminosis D in SLE patients is likely due to the precautions taken by the patients to avoid the sun because of skin photosensitivity, with one study confirming that photosensitivity and photoprotection predicted vitamin D insufficiency and deficiency respectively (87). The use of corticosteroids and antimalarials for SLE treatment can also contribute to reduced vitamin D levels by inhibiting intestinal absorption of cholecalciferol and promoting vitamin D catabolism (90–92). Despite a paucity of studies on JSLE, the existing studies show similar results to adult SLE studies (93–96). Like the adult disease, vitamin D deficiency is prevalent in JSLE patients. This was shown in the APPLE trial which found that 69% of 201 JSLE patients had vitamin D insufficiency and 30% had vitamin D deficiency (93).

As for the relationship between dietary intake and SLE risk, the Nurses’ Health Study (NHS), a prospective cohort study with 186,389 women follow-up for 22 years, reported no relationship between vitamin D intake and risk of SLE (45). Nor was intake in adolescence related to SLE risk in adulthood (97). Similarly, Lourdudoss et al. did not find any protective effect of dietary vitamin D intake on lupus activity in 111 SLE patients during a 2-year period (98).

## Vitamin D Levels and Disease Activity: Therapeutic Implications for SLE and JSLE

As with RA, a number of studies have reported an inverse association between serum 25(OH)D levels and disease activity as measured by the SLEDAI score in the majority of studies, and also by the European Consensus Lupus Activity Measurement – ECLAM score, in one study (99). They are summarized in **Table 4**. In JSLE, one study has also reported a potential inverse relationship between disease activity and 25(OH)D levels (94). Notably, approximately 81% of adult SLE patients also present with fatigue, which remains one of the most troublesome and common symptoms in SLE patients and is inadequately addressed by current treatment strategies (103). Thus, it is important to highlight that two studies have suggested a potentially inverse association between 25(OH)D levels and fatigue scores (87, 102) (**Table 4**). Like RA, most studies were cross-sectional, thus only correlations were inferred. A causal relationship remains undetermined. Importantly, these studies were conducted in different countries around the world, but all reached the same conclusion which shows that hypovitaminosis D in SLE patients is prevalent. This suggests that changes to geographical sunlight levels does not influence these results.

**Table 4:** Impact of vitamin D concentration on JSLE and adult-onset SLE outcomes.

Author et al., year	Country Type of study	Number of participants, F:M, Age	Disease duration	Impact on disease outcomes
**Adult-onset SLE**				
Amital et al., 2010 (99)	Several European and Israeli cohorts Cross-sectional	378 SLE patients 11.1:1 Age – N/A	N/A	Negative correlation between 25(OH)D levels and SLEDAI and ECLAM scores (r = −0.12, p = 0.018)
Borba et al., 2009 (88)	Brazil Cross-sectional	36 SLE patients 26 controls All females 29.6 ± 8.8 - patients 30.0 ± 7.0 - controls	N/A	Multiple regression analysis showed that 25(OH)D levels associated with SLEDAI (r = -0.58). 60% of 25(OH)D variation was attributed to SLEDAI.
Mok et al., 2012 (89)	China Cross-sectional	290 SLE patients 17.1:1 38.9 ± 13.1	7.7 ± 6.7 years	25(OH)D levels significantly inversely correlated with SLEDAI (β = -0.19; p = 0.003)
Yap et al., 2015 (100)	Australia Prospective cohort study 12 months	119 SLE patients 5.3:1 42.2 ± 14.8	8.7 ± 7.1 years	25(OH)D levels significantly inversely correlated with SLEDAI-2K (β = -1.1, p = 0.01). Over 12 months, an increase in vitamin D levels was associated with reduced disease activity.
Sun et al., 2019 (101)	China Cross-sectional	95 SLE patients; 40 controls; 8:1 31.54 ± 11.52 patients 31.20 ± 12.97 controls	N/A	25(OH)D was significantly lower in active SLE patients compared to inactive SLE patients (p < 0.01) which was significantly lower than controls (p < 0.01).
Stockton et al., 2012 (102)	Australia Cross-sectional	24 SLE patients, 21 controls, all women 39.6 ± 11.4 patients 40.9 ± 13.3 controls	N/A	Fatigue scores were significantly higher (p < 0.0001) and muscle group strengths were significantly lower in SLE patients than controls. However, no correlation between fatigue and 25(OH)D levels.
Ruiz-irastorza et al., 2008 (87)	Spain Cross-sectional	92 SLE patients 9.2:1 41 ± 18	7 (0–30) years	Patients with 25(OH)D levels <10 ng/ml had higher but insignificant fatigue scores than those with >10 ng/ml. No correlation between fatigue and 25(OH)D levels.
**JSLE**				
Stagi et al., 2014 (94)	Italy Cross-sectional	45 JSLE patients 109 controls; 4:1 18.9 ± 6.3 patients 17.7 ± 7.4 controls	N/A	Patients with active disease had lower 25(OH)D than those with inactive disease.

ECLAM score, European Consensus Lupus Activity Measurement score; JSLE, juvenile systemic lupus erythematosus; SLE, systemic lupus erythematosus; SLEDAI, systemic lupus erythematosus disease activity index; SLEDAI 2K, systemic lupus erythematosus disease activity index 2000 developed as a modification of SLEDAI in order to include persistent, active disease in those descriptors that had previously only considered new or recurrent occurrences; N/A, not available; 25(OH)D, 25-hydroxy vitamin D.

Regarding the investigation of the vitamin D supplementation role in SLE, most studies reported no significant improvements in disease activity (**Table 5**). Only one study reported a reduced risk of achieving a moderately-high SLEDAI score (≥ 5) after vitamin D supplementation (106). On the other hand, two JSLE demonstrated decreases in disease activity after supplementation (**Table 5**). Notably, most studies either solely or mainly supplemented vitamin D insufficient patients (95, 105–109). Two studies treated both vitamin D insufficient and sufficient patients (96, 104).

**Table 5:** Impact of vitamin D supplementation on JSLE and adult-onset SLE outcomes.

Author et al., year	Country Type of study Study duration	Number of patients vs. control group (if any) F:M Age (mean +/- SD)	Vitamin D dose	Impact on disease activity
**Adult-onset SLE**				
Andreoli et al., 2015 (104)	Italy Prospective study with a cross-over design 24 months	34 SLE patients All female 32.5 (19–44)	Standard regimen (25,000 IU vit D_3_ monthly for one year) then intensive regiment (300,000 IU initial bolus then 50,000 IU a second year), or vice versa	No significant difference in disease activity between standard regimen and intensive regimen.
Karimzadeh et al., 2017 (105)	Iran RCT 12 weeks and 3 months	45 SLE patients in intervention arm vs. 45 in placebo arm 9:1 33.78 ± 6.2 vs. 35.69 ± 6.8	50,000 IU/week vit D_3_ for 12 weeks then 50,000 IU/month for 3 months	No significant difference in SLEDAI in intervention group and placebo group.
Petri et al., 2013 (106)	US Longitudinal observational study Over 128 weeks	763 SLE patients with supplementation vs. 243 SLE patients without supplementation 10.7:1 49.6 ± 13.2	Ergocalciferol 50,000 IU/week and 200 IU of calcium/D_3_ twice daily	Patients with supplementation had a 20 unit increase in 25(OH)D and 21% decrease in the odds of having a high SLEDAI score (≥ 5)
Ruiz-Irastorza et al., 2010 (107)	Spain Longitudinal observational study 2 months	80 SLE patients 9:1 43 ± 14	Varying dosage of oral vit D_3_ 400–1,200 IU/day for 7–24 months	No significant correlations between change in SLEDAI and change in 25(OH)D levels
Terrier et al., 2012 (108)	France Prospective study 4 weeks and 6 months	20 SLE patients All female 31 ± 8	100,000 IU/week vit D_3_ for 4 weeks, then 100,000 IU/month vit D_3_ for 6 months	No significant change in SLEDAI
Zheng et al., 2019 (109)	Asia, South America, North America, Africa Meta-analysis of RCTs	5 RCTs 490 SLE patients	Varying vit D_3_ supplementation	No significant difference in SLEDAI scores.
**JSLE**				
AlSaleem et al., 2015 (96)	Saudi Arabia Cross-sectional 3 months	28 JSLE patients 13:1 9.7 ± 3.2	2,000 IU/daily vit D_3_	An inverse correlation between 25(OH)D and SLEDAI was observed, but it was not statistically significant.
Lima et al., 2016 (95)	Brazil RCT 6 months	20 treatment vs 20 placebo All female 18.5 ± 3.5 vs 19.3 ± 3.3 75	50,000 IU/week vit D_3_	A significantly decrease in SLEDAI and fatigue severity score.

JSLE, juvenile systemic lupus erythematosus; SLE, systemic lupus erythematosus; SLEDAI, systemic lupus erythematosus disease activity index; 25(OH)D, 25-hydroxy vitamin D.

## Immunomodulatory Mechanisms of Vitamin D in Systemic Lupus Erythematosus and JSLE

**Figure 3:**
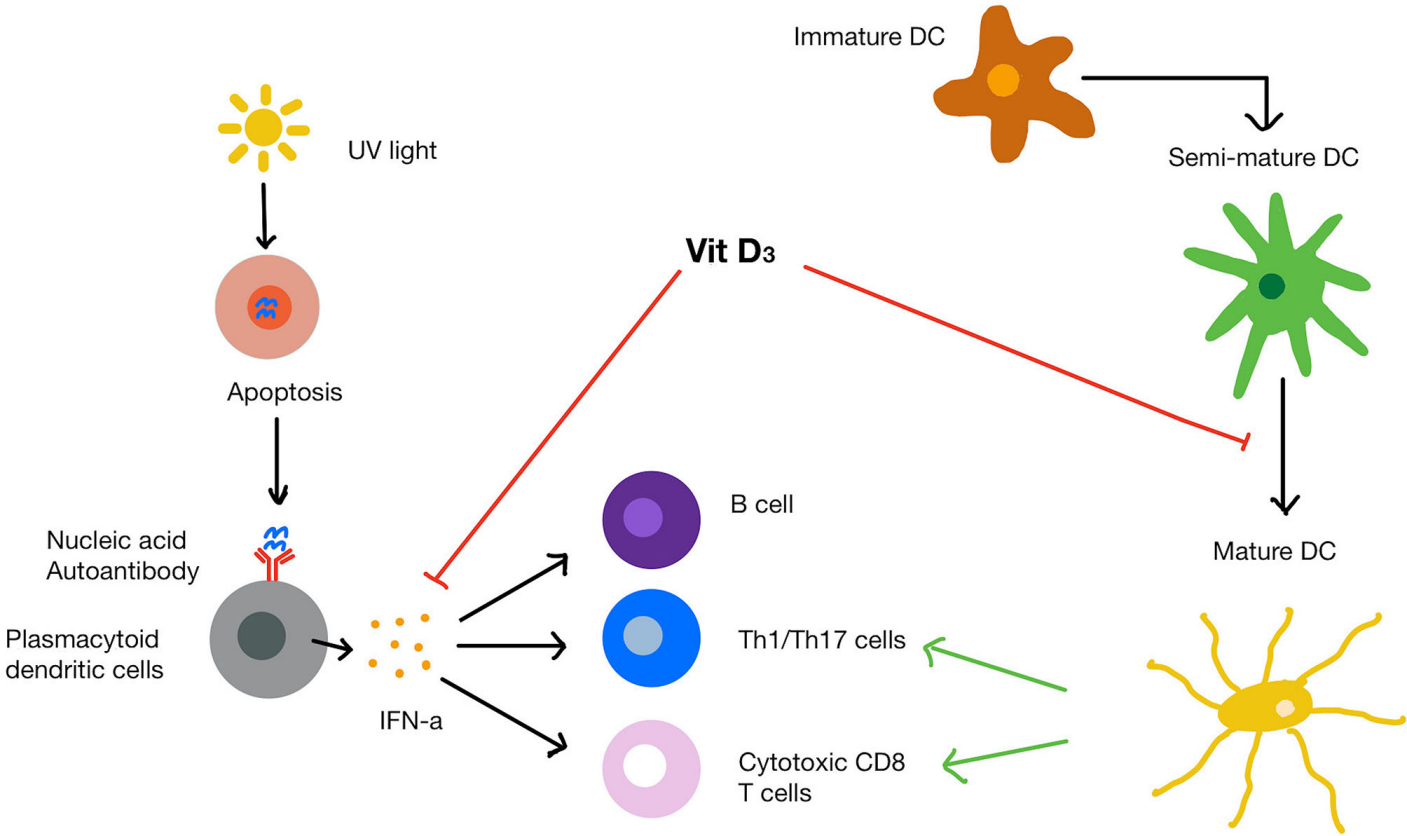
SLE pathogenesis and mechanism of vitamin D on immune cells. UVB light causes apoptosis of cells that release endogenous nucleic acids. The nucleic acids bind to and trigger plasmacytoid dendritic cells to release IFN-α. Mature dendritic cells recruit T helper cells, cytotoxic T cells and B cells. Vitamin D increases protective innate immune response while dampening the over-active immune response. Vitamin D also inhibits the maturation of dendritic cells and suppresses IFN-α production from plasmacytoid dendritic cells as well as IFN-α induced gene expression.

Vitamin D has been demonstrated to influence many immunological pathways that contribute to SLE pathogenesis (**Figure 3**). For example, 1,25(OH)_2_D_3_ has been demonstrated to inhibit of dendritic cell maturation. In one seminal study, VDR knockout mice were shown to have an expansion in mature dendritic cells compared to the wild-type mice (110). In this study, the authors concluded that 1,25(OH)_2_D_3_ can physiologically inhibit dendritic cell maturity even when dendritic cells are exposed to stimuli that usually control their maturation. Similar results have been reported in SLE patients (111), where monocyte-derived dendritic cells responded to 1,25(OH)_2_D_3_ supplementation at 10 nM, which led to a shift towards a semi-mature, tolerogenic phenotype.

Additionally, there is evidence that 1,25(OH)_2_D_3_ can inhibit the expression of IFN-α inducible genes resulting in a reduced IFN signature response (111). A cross-sectional study found that 25(OH)D levels inversely correlated with serum IFN-α levels (r = -0.43, p < 0.0001) and IFN-α gene expression (r = -0.45, p < 0.0001) in SLE patients (112). SLE patients with low serum 25(OH)D levels (≤ 20 ng/ml) overexpressed 2 IFN-α inducible genes compared to those with sufficient serum levels (≥ 20 ng/ml) (16). The expression of these two genes was two-fold lower after cholecalciferol supplementation. However, there are conflicting results as in a subsequent study, Aranow and colleagues did not find any improvement of vitamin D supplementation on IFN signature (113).

1,25(OH)_2_D_3_ has also been shown to suppress the proliferation and differentiation of B cells into memory cells or plasma cells and induce anti-inflammatory cytokine production (IL-10) by regulatory B cells (114). Importantly, there is a well-documented imbalance in these B subsets in SLE patients, with an increase in plasma cells, which contribute to autoantibody production, and a defective regulatory B cell compartment (115). Collectively, these data suggest that the ability of 1,25(OH)_2_D_3_ to skew B cell differentiation in favour of a regulatory phenotype could be efficacious in suppressing inflammation in SLE patients. Although there are limited studies investigating whether vitamin D affects pathogenic mechanisms in SLE, based on the available evidence *in vitro* that vitamin D is able to prevent dendritic cell maturation, inhibit IFN-α production, alter B cell phenotype and potentially reduce the IFN signature response it is likely to have a significant impact on the dysregulated immune response. This represents a serious knowledge gap which should be addressed with future studies.

## Conclusion

RA, JIA, SLE, and JSLE are ARD driven by a dysregulated innate and adaptive immune system. Current treatments aim to control and relieve the symptoms, as well as prevent long-term damage. Nonsteroidal anti-inflammatory drugs (NSAIDs), corticosteroids, disease-modifying anti-rheumatic drugs (DMARDS), and biologic agents are all part of the therapeutic armamentarium for arthritis and lupus. Importantly, many of these drugs are associated with serious side effects such as an increased risk of infection and malignancy, as well as high socioeconomic costs for health services. While no cure remains for these diseases, a significant unmet need remains for cost-effective therapies with limited side effects.

Hypovitaminosis D is prevalent among patients with inflammatory arthritis and SLE across all ages. The majority of studies have so far provided evidence for an inverse association between vitamin D levels and disease activity in RA and adult SLE. The limited number of studies in JIA and JSLE revealed potential associations between the two, but further research is required to confirm these results. Since most studies were cross-sectional, no causal relationship can be inferred, highlighting the necessity for longitudinal studies that investigate the association between vitamin D levels and disease activity within the same patient over time.

In terms of the therapeutic effect of vitamin D supplementation and suppression of disease activity, randomized controlled trials have yet to provide evidence for its efficacy in both diseases. Notably, the studies included in this review have exposed several limitations of the research on this topic. One prominent problem lies in the high heterogeneity between studies regarding the subject population, vitamin D supplementation dose and frequency of administration, outcome measurements, and even definitions of vitamin D deficiency. Trials have been conducted in over 20 countries in various continents, and many found that the prevalence of low vitamin D levels varied geographically, which could be due to differences related to ethnic background, sun exposure, and clothing style. Developmental status of the country, which significantly influences the quality of diet, and therefore vitamin D intake, will also affect vitamin D levels. In addition, the studies investigating the impact of vitamin D supplementation on the risk of developing arthritis or SLE are limited by the use of self-reported and retrospective data. Furthermore, the lack of consensus in dosage regimens between different studies in both RA and SLE means that there is no conclusive evidence of the role of vitamin D supplementation on disease outcomes.

Nevertheless, immunomodulatory studies have offered promising results. *In vitro* addition of 1,25(OH)_2_D_3_ altered the phenotype of dendritic cells, shifted the balance of T helper cells, inhibited pro-inflammatory cytokines and interferon proteins production, and subsequently affected both T and B cell activity, which could be relevant for the pathogenesis of both RA and SLE. Importantly, the wide-ranging effects of vitamin D on potential pathogenic pathways highlights a potential potent immunosuppressive function in these diseases, if the right therapeutic protocol is identified.

Although the scope of this review was to specifically explore the immunomodulatory role of vitamin D in arthritis and lupus across age, there is evidence of its benefits in other ARDs, such as undifferentiated connective tissue disease (UCTD). A 5-week course of daily alfacalcidol supplementation at various doses administered in two open label trials to patients with UCTD restored the capacity of regulatory T cells to suppress the proliferation of autologous CD4+ T cells implicated in the pathogenesis of the disease (116, 117), providing evidence for a potential therapeutic role of vitamin D in the management of this condition. A large study of 1,029 patients with various ARDs, including RA, SLE, autoimmune myositis, scleroderma, antiphospholipid syndrome, multiple sclerosis, and autoimmune thyroid disease, measured the serum vitamin D levels using an immunoluminometric assays and found suboptimal levels (< 20 ng/ml) in all patients (118). This study found no correlation between vitamin D level and SLE disease activity measured using the ECLAM score. Overcoming the limitations of available research in this field would close the gap between hypothesis and treatment efficacy. Future research should focus on large randomized, controlled trials that investigate different doses of vitamin D supplementation at different frequencies of administration, which should also include adequate stratification of patients based on age, disease activity and background medication. The future calls for better designed research including appropriate collection of metadata which allows confounders to be built into multivariate regression models that allow a better understanding of the relationship between vitamin D levels and disease outcomes. To help reduce the study heterogeneity, a clear definition for vitamin D deficiency/insufficiency should be agreed on for both adults and children. Further recommendations for clinical practice should be guided by good quality evidence of the impact of vitamin D supplementation on disease outcomes, as well as additional health benefits (related or unrelated to the underlying ARD). If beneficial, vitamin D has the advantage of being a relatively cheap and relatively safe treatment option. In conclusion, although the level of evidence for the benefits of vitamin D supplementation on outcome measures in arthritis and SLE across ages is poor, the hypothesis of a potential role of vitamin D as an adjuvant treatment option for these diseases remains promising based on available immunological data, but requires further research.

## Author Contributions

JZ, ER, and CC wrote the manuscript. JZ searched the literature and extracted relevant data. All authors contributed to the article and approved the submitted version.

## Funding

This work is supported by a fellowship awarded by the Medical Research Foundation to ECR (MRF-057-0001-RG-ROSS-C0797). ESC was funded by a Barts Charity Lectureship (grant MGU045). CC is supported by NIHR UCLH Biomedical Research Centre (BRC525/III/CC/191350). This work was performed within the Centre for Adolescent Rheumatology Versus Arthritis at UCL, UCLH, and GOSH supported by grants from Versus Arthritis (21593 and 20164), GOSCC, and the NIHR-Biomedical Research Centres at both GOSH and UCLH. The views expressed are those of the authors and not necessarily those of the NHS, the NIHR or the Department of Health.

## Conflict of Interest

The authors declare that the research was conducted in the absence of any commercial or financial relationships that could be construed as a potential conflict of interest.

## Abbreviations

ANA, Anti-nuclear Antibodies; ARD, Autoimmune Rheumatic Diseases; CCP, anti-Cyclic Citrullinated Peptide; CD4, cluster of differentiation 4; DMARD, disease-modifying anti-rheumatic drug; DAS28, Disease Activity Score-28; ECLAM, European Consensus Lupus Activity Measurement; ESR, Erythrocyte Sedimentation Rate; HLA, Human Leukocyte Antigen; JADAS, Juvenile Arthritis Disease Activity Score; JIA, Juvenile Idiopathic Arthritis; JSLE, Juvenile Systemic Lupus Erythematosus; MMP, Matrix Metalloprotease; NSAID, Nonsteroidal anti-inflammatory drug; OPG, Osteoprotegerin; RA, Rheumatoid Arthritis; RANKL, Receptor Activator of Nuclear factor Kappa-B Ligand; RCT, Randomized Controlled Trial; RF, Rheumatoid Factor; RXR, Retinoid X Receptor; SLE, Systemic Lupus Erythematosus; SLEDAI, SLE disease activity index; Th, T helper; VAS, Visual Analogue Scale; VDR, Vitamin D Receptor; VDRE, Vitamin D Receptor Elements; UCTD, undifferentiated connective tissue disease.
